# A genome-wide SNP-based genetic map and QTL mapping for agronomic traits in Chinese cabbage

**DOI:** 10.1038/srep46305

**Published:** 2017-04-18

**Authors:** Li Huang, Yafei Yang, Fang Zhang, Jiashu Cao

**Affiliations:** 1 Laboratory of Cell & Molecular Biology, Institute of Vegetable Science, Zhejiang University, Hangzhou 310058, China; 2 Key Laboratory of Horticultural Plant Growth, Development and Quality Improvement, Ministry of Agriculture, Hangzhou 310058, China; 3 Zhejiang Provincial Key Laboratory of Horticultural Plant Integrative Biology, Hangzhou 310058, China

## Abstract

The aim of this work was to construct a high-resolution genetic map for the dissection of complex morphological and agronomic traits in Chinese cabbage (*Brassica rapa* L. syn. *B. campestris*). Chinese cabbage, an economically important vegetable, is a good model plant for studies on the evolution of morphologic variation. Herein, two high-generation inbred Chinese cabbage lines, ‘Huangxiaoza’ and ‘Bqq094-11’, were crossed. Then restriction-site-associated DNA sequencing (RAD-seq) was performed on the parents and 120 F_2_ individuals. A genetic map containing 711 bins representing 3985 single nucleotide polymorphism (SNP) markers was constructed. By using WinQTL with composite interval mapping (CIM) and mixed-model based composite interval mapping (MCIM) analysis via QTLNetwork, quantitative trait loci (QTL) linked to 16 genetic traits related to plant size, color and leaf characteristics were mapped to 10 linkage groups. The high density genetic map and QTL identified for morphological and agronomic traits lay the groundwork for functional gene mapping, map-based cloning and marker-assisted selection (MAS) in Chinese cabbage.

Brassica is one of the most important cruciferous genera of the Brassicaceae family, and contains more than 40 species^1^,^2^. Among these, Chinese cabbage is one of the most important vegetable crops in China with a long history of cultivation, and diverse cultivars with a variety of qualities and distinct morphological features. With the continual improvement in living standards in China, people are paying increasing attention to the appearance and qualities of Chinese cabbage. This has led to increased attention on future breeding needs for the improvement of its visual appearance and flavor. Thus, understanding the genetic basis of morphological and agronomic traits in Chinese cabbage has become increasingly important.

Most morphological and agronomic traits of Chinese cabbage are quantitative traits. As the breeding of Chinese cabbage still relies mainly on conventional breeding techniques, the selection of these quantitative traits has usually been carried out by phenotypic screening and largely depends on grower experience, and the selected traits are readily affected by the external environment. The selection process could be greatly simplified and improved by using marker-assisted selection (MAS) to construct a genetic map of quantitative trait loci (QTL), which would allow identification of the molecular markers closely linked with or co-segregated with target traits, and achieve accurate genotype detection^3^,^4^.

In recent years, with the rapid development of next-generation sequencing (NGS) technologies and bioinformatic methods, crop breeding theory and technology has undergone major changes. Numerous studies on genetic map construction and MAS have been carried out in Brassica crops. However, this type of research on Chinese cabbage is lagging behind. Before genomic information became available, the markers used in the construction of genetic maps in Chinese cabbage were mostly PCR-based, small in number, and inaccurate, preventing precise QTL analysis of complex quantitative traits. Even after the publication of the Chinese cabbage genome sequence^2^, the number of specific markers remains very limited, and most published markers at present are still PCR-based amplified polymorphism markers such as simple sequence repeats (SSR) and sequence-related amplified polymorphisms (SRAP)^5^,^6^,^7^,^8^,^9^,^10^. Sequence-specific markers that can be used as anchors in Chinese cabbage chromosomes and can be easily applied to genomic mapping are even rarer. Thus, a great deal of work is still needed to improve the genetic map density and QTL mapping accuracy by using high-throughput sequencing technologies and making full use of the reference Chinese cabbage genome. In addition, the currently available completed genomic sequences of Chinese cabbage are estimated to cover only about 60% of the genome. A high-density genetic map with sequence information will also provide an important basis for future genome splicing and assembly^11^,^12^.

Reduced-representation sequencing is an NGS method designed to sequence specific regions of the genome aimed at simplifying genome complexity. It is an effective technology for obtaining large numbers of molecular markers. Restriction-site-associated DNA sequencing (RAD-seq), a representative type of this approach, is widely used for high-throughput single nucleotide polymorphism (SNP) discovery and for genotyping in different organisms^13^. RAD tags are short DNA tags which form a genome-wide representation of every site of a particular restriction enzyme. As most organisms have large numbers of SNPs that disrupt restriction sites, this allows RAD tags to serve as genetic markers spread at a high density throughout the genome^14^.

In this study, using a pair of Chinese cabbage parental cultivars with significantly different morphological traits and their F_2_ progeny as materials, we applied RAD-seq to obtain a large number of SNP markers. Co-segregating SNPs were assigned into bins to build the first high-density genetic map entirely composed of sequencing tags for Chinese cabbage. On this basis, QTL for 16 important morphological and agronomic traits of Chinese cabbage were mapped to 10 chromosomes (linkage groups), laying the foundation for accelerating MAS breeding in Chinese cabbage.

## Results

### The morphological and agronomic traits of Chinese cabbage showed a normal distribution in the F_2_ population and significant positive correlations

**Figure 1:**
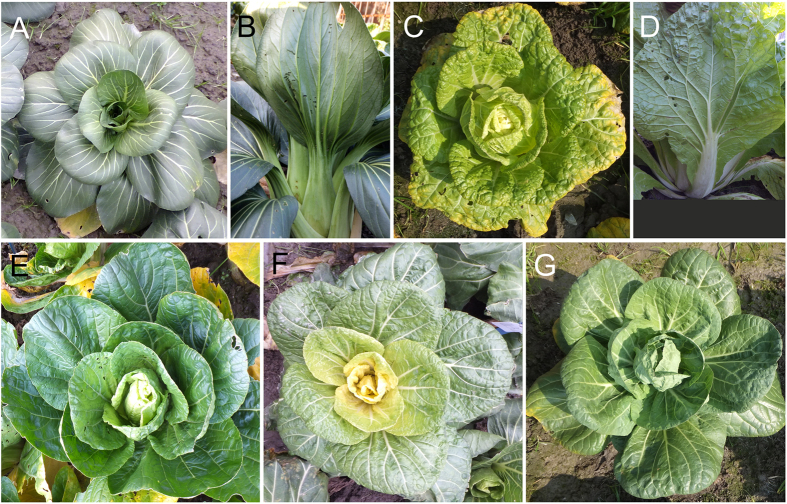
The phenotype of parents and representative F_2_ individuals of Chinese cabbage. (**A**) and (**B**) represent the female parent ‘Bqq094-11’; (**C**) and (**D**) show the phenotypes of male parent ‘Huangxiaoza’; (**E**–**G**) show the phenotypes of representative F_2_ individuals.

To perform this study, we analyzed important morphological and agronomic traits of Chinese cabbage, including plant height, plant diameter, leaf length, leaf width, petiole length, petiole width, petiole with leaf length, leaf wing, leaf shrink, leaf color, heading and bolt length. To identify the loci controlling these features, a mapping population including 120 F_2_ individuals was created from the hybridization of the two parents, high generation inbred lines ‘Huangxiaoza’ and ‘Bqq094-11’ that show different phenotypes for these target traits. ‘Huangxiaoza’ is a cultivar suited for growth in northern China, which is characterized by heading with a shrunken yellow leaf, and a white petiole and leaf wings. The color of its inner leaf is significantly lighter than that of the outer leaf. ‘Bqq094-11’ is a southern regional non-heading Chinese cabbage, whose leaf is smooth without leaf wings. The whole plant including the leaf and petiole is dark green, with no difference in color between the inner and outer leaves (Fig. 1).

**Figure 2:**
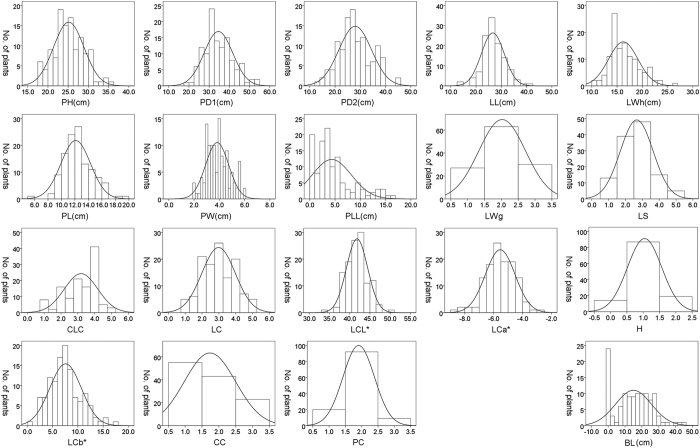
The frequency distribution in the F_2_ population of 16 Chinese cabbage traits. The traits analyzed for the plants are as follows: PH, Plant height; PD1, Plant diameter 1; PD2, Plant diameter 2; LL, Leaf length; LWh, Leaf width; PL, Petiole length; PW, Petiole width; PLL, Petiole with leaf length; LWg, Leaf wing; LS, Leaf shrink; CLC, Core leaf color; LC, Outer leaf color measured by naked eye; LCL*, Outer leaf color measured by CR-300 type colorimeter (from black to white); LCa*, Outer leaf color measured by CR-300 type colorimeter (from green to red) LCb*, Outer leaf color measured by CR-300 type colorimeter b (from blue to yellow); CC, Inner and outer leaf; color contrast; PC, Petiole color; H, Heading; BL, Bolt length.

With the exception of the parental bolt length data, which were missing, the other 15 traits were all found to be significantly different between the two parents (*P* < 0.05) (Table 1). Obvious segregations in the 16 traits were observed in the F_2_ population, and the absolute values of kurtosis and skewness of each trait were less than 2. In addition, the absolute values of skewness and kurtosis for most of the traits were less than 1 (Table 1). These results are consistent with the typical distribution of quantitative traits, i.e., under the polygene hypothesis, the frequency distribution of the traits in F_2_ generation follows a normal distribution. Additionally, with the exception of those traits recorded by grade values, a continuous distribution of other traits was observed in the F_2_ population, indicating a typical genetic inheritance of quantitative traits (Fig. 2).

Correlative analysis of the 16 traits indicated a varying degree of correlation (Table S1). For example, seven of the 10 morphological traits, including plant height, plant diameter 1, plant diameter 2, leaf length, leaf width, petiole length and petiole width, were significantly correlated with each other at the *P* < 0.01 level. High correlations between color traits were also commonly observed. For example, inner and outer leaf color contrast was strongly correlated with core leaf color and outer leaf color (*P* < 0.01), and highly correlated to outer leaf color b* (*P* < 0.05). Heading and bolt length also were significantly correlated (*P* < 0.05). In addition, correlations among different types of traits were observed. For example, plant height was significantly correlated with core leaf color (*P* < 0.05), and was highly significantly correlated with inner and outer leaf color contrast, petiole color, and heading (*P* < 0.01).

### Construction of a high-resolution genetic map of Chinese cabbage based on high-quality RAD-seq data

The two parents and the 120 F_2_ individuals were used to construct genomic libraries and sent for RAD-sequencing. In summary, 2.6 Gb and 2.8 Gb of raw data containing 13,170,599 and 13,979,075 raw reads were obtained in the female and male parents respectively. A total of 98 Gb of raw data containing 493,384,229 raw reads were obtained in 120 F_2_ offspring. Detailed library information is provided in Table S2. The raw sequencing data was strictly filtered to obtain high quality clean data. A total of 2.4 Gb and 2.56 Gb of clean data were obtained in the female and male parent respectively after filtering, and 91 Gb clean data were obtained in the F_2_ offspring.

The clean data was mapped to the NCBI nucleotide database, and no DNA contamination from other organisms was detected (data not shown). After removing the duplicated reads, the paired-end (PE) reads from the parents and F_2_ offspring were mapped against the Chinese cabbage reference genome. The mapping rate to the reference genome of PE reads obtained from the female and male parents were 75.22% and 70.26%, covering 35.19% and 31.40% of the reference genome respectively. The mapping rates of F_2_ offspring to the reference genome had a mean value of 75.22%. The coverage ranged from 15% to 30%, with an average of 19.79% (Fig. S1A). The average sequencing depth detected by SAMtools showed that the mean coverage of SNP loci in female and male parents was 18.00 and 17.20 times respectively. For the F_2_ individuals, the mean coverage of SNP loci was 7.86 times (Fig. S1B).

SAMtools was used to call SNP genotypes between the parents and a total of 27,885 putative SNPs were discovered. Of these, 1700 SNPs were located on the Scaffold, and 26,185 were chromosomal. We then re-examined the genotypes of these loci in the F_2_ population, and only the genotypes found in more than 85% of progeny (i.e., 102 of the 120 individuals) were further selected. This screening reduced the SNP number to 8,147. After further filtering loci located on the Scaffold and showing significant segregation distortion (*χ*^2^ test, *P* < 0.001), a final set of 4047 SNPs was retained.

**Figure 3:**
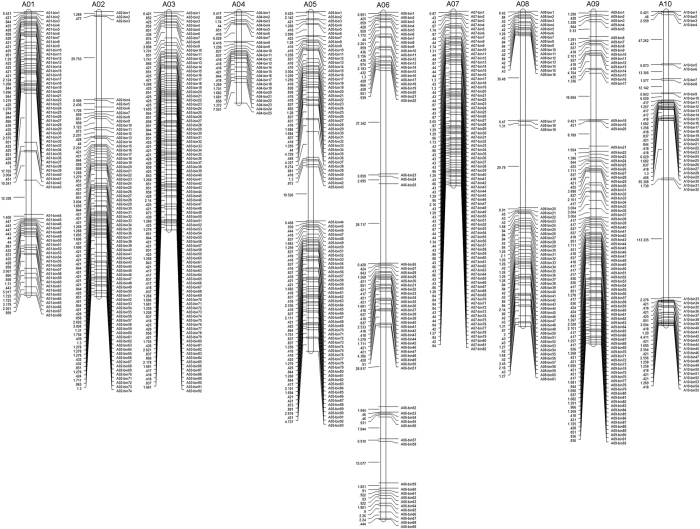
Bin distribution of Chinese cabbage linkage groups. The genetic distance (centimorgans, cM) is shown on the left side of each linkage group, and the marker names are shown on the right side.

Finally, 3985 SNPs were assigned to 711 recombination bins and a high-density genetic map was constructed by mapping these bin markers onto the 10 Chinese cabbage linkage groups (chromosomes) (Fig. 3). The total physical distance of the linkage groups was 255.09 Mb, with an average distance of 358.77 Kb between linkage regions. The total genetic distance of the map was 1223.44 cM, with an average interval of 1.72 cM between adjacent markers/bins. Over 70% of the inter-bin distances (490 bins related) were less than 1 cM. A distance between bin pairs larger than 15 cM was only found in 13 regions, which were dispersed on chromosomes A01 (18 cM), A02 (30 cM), A5 (20 cM), A6 (37, 29, 29, 15 cM), A8 (30, 30 cM), A9 (17 cM), A10 (113, 55, 47 cM), accounting for 1.8% of the total number of bins (Table 2, Table S3).

**Figure 4:**
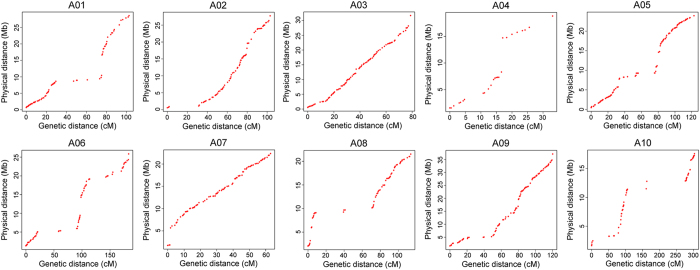
Correlation between genetic distance and physical distance for the ten chromosomes in the Chinese cabbage genome. Genetic distance (cM) is derived from the genetic map, physical distance (Mb) is derived from concatenated scaffolds of *B. rapa* ‘Chiifu-401’.

The collinearity between the constructed genetic map and the Chinese cabbage reference genome was then investigated. It was found that on chromosomes A02, A03, A05, A07, and A09, the correlation between the genetic distance and the physical distance appeared to be almost linear across the entire chromosome, showing no significant intermittent or negative slope. A relatively low correlation between physical distance and genetic distance of chromosomes A01, A04, A06, A08, and A10 was found, though linear trends could be observed (Fig. 4).

### QTL controlling fifteen traits were detected by WinQTL software

On the basis of the high-density RAD genetic map, QTL mapping of the 16 Chinese cabbage traits was conducted. To maximize the mapping accuracy, a two-step procedures involving WinQTL cartographer 2.5 based on a composite interval mapping (CIM) model and QTLNetwork 2.1 via mixed-model-based composite interval mapping (MCIM) method was used to detect the QTL.

**Figure 5:**
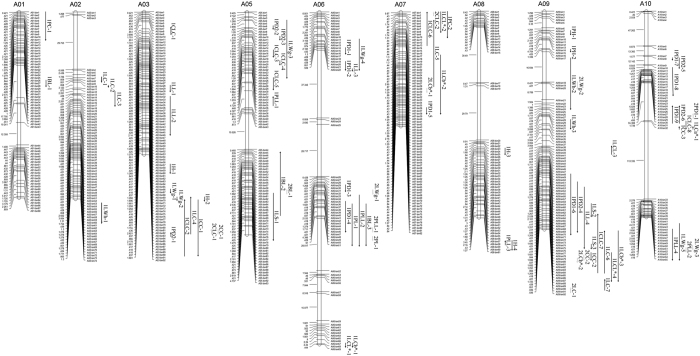
Distribution of QTLs in the Chinese cabbage genetic linkage map. Solid arrows indicate QTL loci identified by WinQTL cartographer 2.5, and hollow arrows indicate QTL loci identified by QTLNetwork 2.1.

By performing CIM analysis using WinQTL, a total of 72 QTL were found for all traits with the exception of the petiole width (Table 3, Fig. 5). Two QTL with negative additive effects on plant height were detected on chromosome A09, accounting for 10.52% and 11.44% of phenotypic variation, respectively. Nine QTL affecting plant diameter 1 were identified; four on chromosome A06, three on chromosome A10, and one each on chromosomes A07 and A09, of which 1PD1-8 located on A10 accounted for the most phenotypic variation (20.99%). Six QTL were found to be related to plant diameter 2, two each located on chromosomes A05 and A10, and one each on chromosomes A03 and A09. Among them, 1PD2-5 on A10 had the largest effect explaining 18.49% of the observed phenotypic variation. Four QTL linked to leaf length were identified on chromosomes A03, A06, and A09, among which, 35.29% of phenotypic variation was explained by 1LL-3 on A06. Two QTL associated with leaf width were detected on chromosome A09 and one on chromosome A02, of which 1LWh-2 on A09 accounted for most of the phenotypic variation (17.78%). One QTL located on chromosome A06 was found to be linked to leaf length, which explained 12.10% of the phenotypic variation. Four QTL mapping on chromosomes A05, A06, A08, and A10 were associated with petiole and leaf length, and had little difference in their genetic contribution (the phenotypic variation explained by the four QTL ranged from 4.86% to 7.88%). Five QTL affecting leaf wing were detected: two on chromosome A03, and one each on chromosomes A05, A06, and A10. In this case, 1LWg-4 located on A06 explained most (18.43%) of the phenotypic variation. As for leaf shrink, three QTL were identified, including one on chromosome A05 and two on chromosome A09. The highest phenotypic variation (16.33%) was explained by 1LS-2 located on A09. Eight QTL associated with core leaf color were targeted to chromosomes A03, A05, A07, A09, and A10. Among these, 1CLC-1 and 1CLC-2 located on A03 explained a total of 30.63% of this trait variation. Seven QTL located on chromosomes A02, A03, A07, and A09 were associated with outer leaf color as observed by the naked eye. These QTL explained 7.78–15.88% of the phenotypic variation, among which 1LC-6 and 1LC-7 located on A09 had a total of 28.35% contribution to the variation. As measured by a CR-300 type colorimeter, four QTL associated with outer leaf color L* were also mapped explaining 6.29–11.28% of the phenotypic variation of this trait. Additionally, chromosome A10 harbored one QTL that affected the outer leaf color a*. This QTL, which showed an additive effect, could explain 80.38% of the phenotypic variation. In addition, three QTL related to outer leaf color b* were identified on chromosomes A06, A07, and A09, of which 1LCb*-3 on chromosome A09 had the largest genetic contribution explaining 22.56% of the phenotypic variation. Three QTL were related to inner and outer leaf color contrast on chromosomes A03, A09, and A10. 1CC-3 on A10 could be the major locus for this trait because it contributed up to 61.3% of the variation. Two QTL with similar contributions (explaining 12.1% and 9.86% of the phenotypic variation respectively) on chromosomes A01 and A07 were detected affecting petiole color. Four QTL, two each on chromosomes A03 and A08, were found associated with heading. The contributions of the two QTL to the phenotypic variation on chromosome A03 were about 13%, and the contributions of the other two QTL located on chromosome A08 were around 10%. As for bolt length, three QTL located on chromosomes A01, A05 and A06 were identified. The phenotypic variations explained by these QTL were all below 10%. It should be pointed out that 22 QTL were identified with logarithm of the odds (LOD) values between 2.5 and 3. These QTL may be unreliable and should be used with caution for further gene mapping or cloning.

### QTL linked to nine traits were detected using QTLNetwork software

By using QTLNetwork to determine the QTL for the 16 traits, a total of 15 QTL were detected associated with eight traits, and two pairs of epistatic QTL were found associated with core leaf color and plant diameter 2 (Tables 4 and 5, Fig. 5). One QTL was detected associated with plant diameter 1, petiole length, outer leaf color, and bolt length, which explained 12.30%, 14.28%, 8.94%, and 15.50% of each trait respectively. Two QTL involved in petiole with leaf length were detected on chromosomes A06 and A10, and accounted for 46.84% of the total phenotypic variation. Three QTL impacting leaf wing located on chromosomes A06, A09 and A10, accounted for up to 50.67% of the variation of this trait. Two QTL related to core leaf color were detected on chromosomes A03 and A07, and their contribution to the phenotypic variation was 50.10%. On chromosomes A07 and A09, two QTL associated with outer leaf color b* explained 27.08% of the phenotypic variation. Two QTL affecting inner and outer leaf color contrast, explaining a total of 25.91% of the phenotypic variation were found on chromosomes A03 and A09.

In addition, one pair of epistatic QTL, associated with the core leaf color, were detected on chromosomes A05 and A09 respectively. Another pair of epistatic QTL located on chromosomes A03 and A09, were associated with plant diameter 2.

### Eleven common QTL were determined by WinQTL and QTLNetwork

As described above, the number of QTL detected through WinQTL was far more than the number detected by QTLNetwork. We compared the outputs of WinQTL and QTLNetwork, and found 11 common QTL controlling nine traits (Table 6). However, the positions of the QTL detected were not entirely consistent; a total of eight QTL differed within 0.1 cM, and two QTL differed between 0.1 cM and 0.5 cM. For QTL in the same position or close to one another, each software gave basically the same value of their contributions to phenotypic variation and additive effects. However, QTLNetwork gave generally higher values overall than WinQTL, in particular the values of explained variation.

It should be noted that four QTL detected by QTLNetwork were not detected by WinQTL as follows: 2PLL-1 associated with petiole with leaf length, 2LWg-1 and 2LWg-2 associated with leaf wing, and 2LCb*-1 with outer leaf color b*. In addition, as WinQTL uses a CIM model to detect QTL, epistatic relationships could not be detected.

## Discussion

The emergence of NGS technology and its wide application have greatly reduced the cost of nucleotide sequencing, although the price remains a consideration when planning sequencing strategies. For some studies, there is no need for whole genome resequencing^15^. Genome complexity can be reduced by restriction enzyme digestion and such simplified genome sequencing technologies are now widely used for large-scale high-throughput SNP genotyping, particularly for *de novo* SNP discovery. In addition to the advantage of high density and high throughput^14^, RAD-seq is a versatile method, which can be used on any number of individuals at any depth of sequencing, depending on available resources^16^. For these reasons, since its inception in 2007, RAD-seq has become one of the main technologies employed to rapidly discover SNPs and has been widely used in many species in recent years^17^,^18^,^19^,^20^,^21^,^22^,^23^,^24^,^25^,^26^.

Here, we used RAD-seq to construct a new genetic map of Chinese cabbage, which already has a reference genome. One major advantage of RAD-seq in species that have a reference genome, is that researchers can pre-select a suitable enzyme to perform an *in silico* digest of the reference genome, so as to estimate the distribution of digested fragments, and thereby know what to expect from the output that will be generated. In this study, we sequenced two parents and 120 F_2_ Chinese cabbage individuals, and obtained a total of 4,047 effective SNP markers. Only the homozygous SNP markers between parents and F_2_-type SNP markers genotyped in more than 85% of progeny were further selected. This selection criterion is much higher than that reported in many other studies^24^,^25^. Therefore, to obtain more SNPs, we suggest that the standard could be reduced further, e.g. 80% genotype coverage of the F_2_ individual.

Linear analysis of the genetic map constructed in this study and the Chinese cabbage reference genome^12^ showed that the genetic distance and physical distance of each linkage group has a significant co-linear trend, indicating that the constructed map has a high credibility. However, there were still some gaps between the genetic and physical distances on chromosomes A01, A04, A06, A08, and A10. The possible causes might be: (1) the assembly size of Chinese cabbage reference genome is still some distance from completion; (2) the linear correlation of markers on the RCZ16_DH map used to guide the Chinese cabbage genome assembly with the assembled sequence is also only about 93%, and there were some irregular distributions on chromosome A02, A04, A06, A08 and A10, similar to our study^12^; and (3) compared with the number of markers obtained by whole genome sequencing, the number of markers obtained by reduced-representation genome sequencing is relatively small, according to studies in other species^27^. It therefore may not be accurate to assess the relationship between genetic distance and physical distance in this case. Similarly, when we looked at the relationship between the Chinese cabbage VCS-13M DH map which was also built via high-throughput sequencing, with the reference genome^28^, we also found some negative slopes and gaps that were even larger than those in our study.

Since Song *et al*. built the first amplified fragment length polymorphism (AFLP) map^29^, more than 30 genetic maps have been constructed for Chinese cabbage^5^,^6^,^7^,^8^,^30^,^31^. Along with the launch of the multinational *B. rapa* Genome Sequencing Project (BrGSP) in 2003, Choi *et al*. constructed a map comprising a total of 556 markers by using the doubled haploid (DH) lines^8^. The total length of this linkage map is 1,182 cM with an average interval of 2.83 cM between adjacent loci, forming the backbone for anchoring sequence contigs for BrGSP. In fact, only the integration of the genetic, physical, and chromosome maps will provide a better guide for crop breeding. Therefore, the first generation physical map and the second generation linkage map were built successively^6^,^30^. Three representative reference linkage maps, which were constructed by the use of SSR markers, are the VCS_DH map^31^, the CK_DH map^6^ and the JWF3P map^5^. However, the genome coverage of these maps is incomplete, anchoring only approximately 73.6% of the scaffolds onto chromosomes. To better improve the assembly of the Chinese cabbage genome, a new map named RCZ16_DH was constructed. This map comprises a total of 507 markers including 415 insertion/deletion length polymorphisms (InDels) and 92 SSRs, 1234.2 cM in total length with an average distance of 2.43 cM between adjacent marker loci^12^. After the release of the Chinese cabbage genome (http://www.Brassica-rapa.org), two linkage maps were constructed. One comprises 503 InDels^32^, and the other comprises 221 SNPs, 31 InDels and 62 SSRs, covering 1,115.9 cM with an average interval of 3.6 cM between the adjacent marker loci^28^.

The genetic maps constructed so far are not saturated enough to perform detailed studies of genetic traits. This is because the populations used in the mapping studies were not large enough or too few genetic markers were used. In this study, we sequenced a population consisting of 120 F_2_ individuals and their two parents, and obtained a set of SNP markers which are more widespread than InDel markers previously used in genetic mapping of Chinese cabbage^33^. A total of 711 bin markers representing 3985 SNP markers were mapped onto 10 linkage groups, covering 1223.44 cM with an average inter-bin distance of 1.72 cM. The number of markers used in this map is far more than any other map built in the past. Meanwhile, markers across linkage groups fit a uniform distribution. This new Chinese cabbage genetic map is the most saturated map by far, and undoubtedly lays the foundation for further genomics and genetic breeding studies.

Knowledge of the genetic basis of variation underlying quantitative traits and QTL mapping is critical for improved breeding of Chinese cabbage. A significant amount of research has been done in Chinese cabbage on QTL detection for traits linked to plant morphology, vernalization, bolting and flowering, disease resistance and yield^34^,^35^,^36^,^37^,^38^,^39^,^40^,^41^,^42^,^43^. However, this is limited by the accuracy of QTL mapping and genetic information in part due to the populations used for genetic studies and the complex genetics of Chinese cabbage. Therefore, to meet the requirements for breeding, QTL mapping of the important morphological and agronomic traits in Chinese cabbage is still a critical issue.

The morphological traits of Chinese cabbage such as plant height, plant width, leaf length, and petiole length are inseparably linked to its yield and appearance. Leaf color is closely related to photosynthesis and consumer preferences. Heading and bolting go together with edible qualities. These traits together determine the characteristics and quality of Chinese cabbage^36^. Previous studies detected several QTL associated with leaf architecture traits including plant height, leaf length, leaf width, and petiole length, in Chinese cabbage^37^,^43^. Some newly detected QTL in our study are located on the same linkage group with those of previous studies but at different positions. For example, QTL affecting leaf length were found on chromosomes A03, A06 and A09 both in our study and the previous studies^37^,^43^. However, there were more novel QTL detected in our study than QTL that were previously identified. Our study also mapped QTL for leaf color for the first time in Chinese cabbage. Most of these are located on chromosomes A03, A06 and A09. The identification of these QTLs for leaf traits will no doubt help us to facilitate the identification of key genes and increase our understanding of the molecular basis of leaf development in Chinese cabbage. Together with the QTL mapping of genes underlying the important traits contributing to heading and bolting, we can further dissect the genetic architecture underlying quantitative traits and accelerate MAS breeding to develop novel high-yielding, high-quality Chinese cabbage varieties.

Knowledge of genetics has indicated that complex significant correlations exist among agronomic traits, probably because of closely linked genes or pleiotropic effects from the same gene. This kind of aggregation of QTL and pleiotropic effects occurs frequently in Chinese cabbage and other crops^37^. In this study, as shown in Fig. 5, many co-located QTL were also detected. For example, according to the results detected by QTLNetwork, 2PL-1 associated with petiole length, 2PLL-1 associated with petiole with leaf length, and 2LWg-1 associated with leaf wing were co-located between bin31 and bin49 on chromosome A06. It is worth mentioning that significant correlations have often been found in traits with the same QTL locations. Similar results were achieved by WinQTL testing, in which considerable overlap among locations of QTL for different agronomic traits corresponded well with the positive correlations usually observed among these sets of phenotypes. These results further suggest that for interconnected or functionally similar traits, their QTL are often located on similar regions or even the same region in the same linkage group. Further fine mapping could help us to determine whether such regions contain a major QTL with pleiotropic effects on several traits or multiple linked QTL^44^. The identification of large-effect pleiotropic or closely linked QTL would be of great value for breeding program where the goal is to simultaneously improve various agronomically important traits.

## Methods

### Plant material and trait measurement

The mapping population we used was 120 F_2_ individuals which came from crossing the high generation inbred lines ‘Huangxiaoza’ (male parent) and ‘Bqq094-11’ (female parent). All the tested materials were grown in the vegetable research farm of Zhejiang University. All traits were surveyed at the normal harvest time (Fig. S2). The standards of measurement were as follows. Plant height (PH): the maximum height of the highest point of the plant from the ground. Plant diameter 1 (PD1): the maximum distance of the outer leaves of the plant. Plant diameter 2 (PD2): the minimum distance of the outer leaves of the plant. Leaf length (LL): the length of the maximum outer leaves from the petiole base to the leaf apex. Leaf width (LWh): the width of maximum outer leaf at its widest point. Petiole length (PL): the length from the base of petioles to the tip of midrib of the largest rosette leaf. Petiole width (PW): the width of petioles at the widest point of the largest outer leaf. Petiole with leaf length (PLL): the length from the tip of midrib to the wing tip at the bottom of the largest rosette leaf. The measurement units of the above characteristics were centimeters (cm). Leaf wing (LWg): according to the largest outer leaves, they were divided into three grades from without wing to with large wing, and those without leaf wing were marked as “1”. Leaf shrink (LS): the wrinkle level of the largest outer leaves. According to the level of wrinkle in the leaves, they were divided into five grades, of which those with a smooth surface were marked as “1”. Core leaf color (CLC): the color of the inner leaves deviated from leaf veins in the center of plant. Leaves were divided into five grades of core leaf color from yellow to green, with yellow marked as “1”, dark green marked as “5”. Outer leaf color (leaf color, LC) was measured using a CR-300 type colorimeter (Minolta, Japan) and the naked eye to measure the color of the largest outer leaf deviated from leaf veins. The colorimeter can respectively evaluate in three-dimensions: L (from black to white, 0 to 100), a (from green to red, −A to +A), b (from blue to yellow, −B to +B). Each plant was measured using three points; 1) as measured by the naked eye from yellow green to dark green plants were divided into five grades, with yellow green marked as “1”, dark green as “5”; 2) the color traits of outer leaves measured by colorimeter were recorded as LCL*, LCa*, LCb*; 3) color of outer leaves measured by naked eye was recorded as LC. Inner and outer leaf color contrast (color contrast, CC): according to the degree of color contrast leaves were divided into three grades, of which those with no color contrast were marked as “1”. Petiole color (PC): the color at the base of petiole at the back of the largest outer leaf. Petiole color was divided into three grades, of which the whitest was marked as “1”, and the greenest was marked as “3”. Heading (H): according to whether the plant headed or not, the heading trait was divided into three grades, non-heading is marked as “1”, and heading as “3”. Bolt length (BL): distance (cm) from the emergence site of the cotyledon to the vertex of the flower stalk. All of the above measurements were repeated three times. For LWg, LS and CC, we preferred to use the value which appeared most frequently in the measurements, and for the other traits, the values were averaged from the three repeats.

### Phenotypic characterizations

The phenotypic data were analyzed using SPSS 18.0 statistical software. To analyze the characteristics of the two parents, we used the “analyze - compare means - independent sample T test” process to assess the difference in parental traits. For the 120 F_2_ individuals, we used the “analyze - descriptive statistics - frequencies” to make a count of variance, range, skewness and kurtosis, and obtained a cylindrical profile. We made a correlation analysis of the 120 F_2_ individuals’ different agronomic traits using the “analyze-correlate-bivariate” process, and account correlation matrix of the Pearson correlation coefficient.

### RAD library construction and sequencing

Genomic DNA from parents and their F_2_ progeny was extracted using the DP320 kit (TIANGEN Biotech Co., Ltd. Beijing, China). The DNA samples of female parent ‘Bqq094-11’ and the male parent ‘Huangxiaoza’ were recorded as 121 and 122 respectively, and those of the 120 F_2_ individuals were numbered from 1 to 120. The DNA was quantified with the Quant-iTTM dsDNA High-sensitivity kit (Invitrogen, Grand Island, NY, USA), so that the starting amount of the parental DNA was 1.5 μg, and that of the progeny was 300 ng. The DNA was digested with *EcoR*I (New England Biolabs, Ipswich, MA, USA), the digestion product samples were ligated to a P1 adapter with the Illumina Solexa kit ©. DNA was processed to obtain RAD libraries and a Covaris S220 ultra-sonicator was used to break DNA to 300–700 bp in length, and 1.6× AMPure XP Beads were used to purify the DNA samples. A Quick Blunting kit Enzyme Mix (New England Biolabs) and Klenow fragment (exo-; New England Biolabs) were used to blunt end and add adenine to the 3′ end. The DNA was then ligated with a P2 adapter, followed by purification using 1.6 × AMPure XP Beads and then used for selective PCR using 2× Phusion PCR Master Mix (NEB) over 18 cycles. PCR products were subjected to agarose gel (1.5%) electrophoresis, and the DNA between 300 bp to 700 bp was extracted and purified by QIAGEN Gel Extraction Kit. Each RAD library was quantified using an Agilent Bioanalyzer (Santa Clara, CA, USA), and finally sequenced on an Illumina HiSeq2000PE100 by Beijing Novogene Bioinformatics Technology Co. Ltd (http://novogene0.bioon.com.cn/).

### Quality control of sequencing data and mapping reads against the reference genome

The output raw sequencing data/reads were filtered to discard those containing adapter contamination, low-quality base reads (Q score ≤ 5) and ambiguous base reads (N). Specifically, reads should not contain adapter sequences; ambiguous bases should not comprise more than 10% of the sequence length, and low-quality bases should not account for more than 50% of the total bases of the read. Then the number of total reads, total data output, overall error rate, Q20 content, Q30 content, and GC content of the clean data were calculated. Contamination with DNA from other sources was assessed by aligning the clean data with the nucleotide databases of National Center for Biotechnology Information (NCBI, http://archive-dtd.ncbi.nlm.nih.gov/). Following removal of duplicates in the clean reads, the paired-end (PE) reads were mapped against the Chinese cabbage reference genome (http://brassicadb.org) using BWA software^45^ applying the following parameters, aln-o 1 -m 100000 -t 4 -l 32 -i 15 -q 10. Any duplicates in the mapping results were removed by SAMtools’ rmdup^46^.

### SNP detection and SNP marker-based genetic map construction

SNPs detection was performed using SAMtools. In order to reduce false positives, we required a minimum threshold of four copies and a maximum threshold of 1000 copies of each allele in the parental samples^47^. In the progeny, the maximum threshold was the same but the minimum threshold was two copies^48^. Only the homozygous SNP markers between parents and F_2_-type SNP markers genotyped in more than 85% of progeny were further selected. Markers showing significant segregation distortion (*χ*^2^ test, *P* < 0.001, d.f. = 2) were excluded^49^,^50^. Co-segregating SNP loci were collapsed into bins, and a linkage bin-map was constructed from the recombination bins serving as genetic markers using ScMap software (Novogene Bioinformatics Technology Co. Ltd, Beijing China). The ScMap software used a modified version of the sliding-window approach developed by Huang *et al*.^51^.

### QTL mapping

QTL mapping was performed with WinQTL cartographer 2.5 and QTLNetwork 2.1^52^,^53^. For QTL analysis with WinQTL cartographer 2.5, a permutation test (parameter 1000) was used to determine the QTL with a LOD threshold of 2.5. CIM was performed using Model 6, a value of 5 for control markers and a forward regression method, scanning intervals of 1 cM between markers and putative QTL with a window size of 10 cM. A 1000 permutation test at 95% confidence level to determine the LOD thresholds, with significance set as *P* < 0.05^54^. By using QTLNetwork v2.1 (http://ibi.zju.edu.cn/software/qtlnetwork/download.htm) and a MCIM method, QTL was determined by a 1,000-permutation test at the confidence level of 95%. The window size and walk speed used for the genome scan were 10 cM and 1 cM, respectively. The QTL identified were named as 1/2 followed by trait abbreviation and QTL number, in which 1 represents the QTL detected by WinQTL cartographer 2.5, and 2 represents the QTL detected by QTLNetwork 2.1. For example, 1PH-1 represents the first QTL site of plant height detected by WinQTL cartographer 2.5.

## Additional Information

**How to cite this article:** Huang, L. *et al*. A genome-wide SNP-based genetic map and QTL mapping for agronomic traits in Chinese cabbage. *Sci. Rep.* **7**, 46305; doi: 10.1038/srep46305 (2017).

**Publisher's note:** Springer Nature remains neutral with regard to jurisdictional claims in published maps and institutional affiliations.

## Supplementary Material

Supplementary Dataset 1

Supplementary Dataset 2

## Figures and Tables

**Table 1:** Parental values and distribution in the F_2_ population for 16 Chinese cabbage traits.

Traits	Parents values		Distribution in the F_2_ population			
	‘Bqq094-11’	‘Huangxiaoza’	SD	Range	Skewness	Kurtosis
PH	25.7A	22.0 B	3.769	18.00	0.29	−0.05
PD1	39.2A	34.4 B	7.068	35.70	0.39	−0.08
PD2	33.5 a	29.9 b	6.448	30.60	0.22	−0.33
LL	28.4A	23.6 B	4.533	27.50	0.06	1.07
LWh	18.6A	13.9 B	2.911	15.50	0.72	0.37
PL	12.8A	8.5 B	2.193	14.50	0.34	0.98
PW	4.6A	6.0 B	0.909	4.00	0.33	−0.41
PLL	12.8A	0 B	3.897	16.00	1.20	0.87
LWg	3A	1 B	0.692	2.00	−0.03	−0.88
LS	5A	1 B	0.973	4.00	0.27	−0.08
CLC	1A	5 B	0.996	4.00	−0.61	−0.407
LC	1A	5 B	0.995	4.00	0.06	−0.65
LCL^*^	44.79A	39.66 B	2.636	16.78	0.23	0.53
LCa^*^	7.16A	3.25 B	1.030	5.82	−0.29	0.35
LCb^*^	14.53A	3.55 B	3.144	17.00	0.55	0.28
CC	3A	1 B	0.761	2.00	0.48	−1.13
PC	1A	3 B	0.478	2.00	−0.25	1.30
H	2A	0 B	0.525	2.00	0.05	0.71
BL	—	—	10.678	44.50	0.19	−0.43

Note: PH, Plant Height; PD1, Plant Diameter 1; PD2, Plant Diameter 2; LL, Leaf Length; LWh, Leaf Width; PL, Petiole Length; PW, Petiole Width; PLL, Petiole With Leaf Length; LWg, Leaf Wing; LS, Leaf Shrink; CLC, Core Leaf Color; LC, Leaf Color; LCL*, Outer leaf color measured by CR-300 type colorimeter (from black to white); LCa*, Outer leaf color measured by CR-300 type colorimeter (from green to red); LCb*, Outer leaf color measured by CR-300 type colorimeter b (from blue to yellow); CC, inner and outer leaf Color Contrast; PC, Petiole Color; H, Heading; BL, Bolt Length. SD, Standard Deviation. Lowercase letters mean significant difference (P < 0.05) and capital letters mean significant difference (P < 0.01).

**Table 2:** Characteristics of the linkage groups in the maternal genetic map of Chinese cabbage.

LG	PD (Mb)	GD (cM)	SNP NO.	Bin NO.	APD (Kbp)	AGD (cM)
A01	28.58	102.55	292	68	420.29	1.51
A02	27.72	103.04	390	74	374.59	1.39
A03	31.68	78.80	405	92	344.35	0.86
A04	18.80	33.10	223	25	752.00	1.32
A05	23.93	122.82	593	93	257.31	1.32
A06	25.85	183.26	290	69	374.64	2.66
A07	22.36	62.97	467	82	272.68	0.77
A08	21.52	113.73	377	61	352.79	1.86
A09	37.08	120.32	660	92	403.04	1.31
A10	17.56	302.85	288	55	319.27	5.51
Min value	17.56	33.10	223	25	702.40	0.77
Max value	37.08	302.85	593	93	398.71	5.51
total	255.09	1223.44	3985	711	358.77	1.72

Note: LG, linkage group; PD, Physical distance; GD, Genetic distance; APD, Average physical distance; AGD, Average genetic distance.

**Table 3:** QTL identified in the F_2_ population of Chinese cabbage using WinQTL cartographer 2.5.

Traits	QTL name	LG	Bin interval	Position (cM)	AE	LOD value	EV (%)
PH	1PH-1	9	7~8	10.87	−1.779	**2**.**93**	10.52
	1PH-2	9	14	15.90	−1.853	**2**.**73**	11.44
PD1	1PD1-1	6	14~16	15.79	−2.144	3.18	4.06
	1PD1-2	6	19~22	19.24	−2.716	**2**.**72**	6.73
	1PD1-3	6	29~31	93.61	−2.055	**2**.**74**	4.61
	1PD1-4	6	35~46	98.24	−2.841	3.20	8.08
	1PD1-5	7	36~38	29.96	−3.080	3.02	8.94
	1PD1-6	9	48~70	89.50	−3.635	4.47	14.50
	1PD1-7	10	8	73.47	−4.386	**2**.**89**	15.30
	1PD1-8	10	10~20	88.41	−6.018	4.33	20.99
	1PD1-9	10	24~30	103.25	2.093	5.69	2.42
PD2	1PD2-1	3	82~83	69.47	3.049	**2**.**91**	11.78
	1PD2-2	5	6~7	8.58	−2.291	**2**.**69**	5.84
	1PD2-3	5	10~11	11.12	−2.232	**2**.**84**	5.47
	1PD2-4	9	51~69	89.08	−3.304	4.14	14.74
	1PD2-5	10	8~10	74.47	−4.417	3.45	18.49
	1PD2-6	10	24~30	103.25	2.305	4.17	4.22
LL	1LL-1	3	28~30	26.51	−2.085	**2**.**70**	10.80
	1LL-2	3	32~46	34.14	−2.348	3.54	14.34
	1LL-3	6	22	36.03	−4.394	**2**.**54**	35.29
	1LL-4	9	53~75	94.14	−2.336	4.31	15.36
LWh	1LWh-1	2	53~60	87.12	−0.935	3.43	5.36
	1LWh-2	9	17~24	45.90	−1.723	4.13	17.78
	1LWh-3	9	28~31	57.24	−1.454	**2**.**87**	12.65
PL	1PL-1	6	36~51	108.95	−1.126	4.24	12.10
PLL	1PLL-1	5	32~35	33.45	2.912	3.42	6.36
	1PLL-2	6	33~51	126.68	−1.294	6.35	4.86
	1PLL-3	8	57~58	109.88	1.361	**2**.**94**	6.57
	1PLL-4	10	44~54	300.33	−1.603	9.83	7.88
LWg	1LWg-1	3	66~67	52.44	−0.238	**2**.**80**	6.02
	1LWg-2	3	69~70	55.11	−0.273	**2**.**64**	7.68
	1LWg-3	5	4~25	10.71	−0.255	3.45	6.24
	1LWg-4	6	11~22	17.95	−0.436	5.72	18.43
	1LWg-5	10	42~54	299.65	−0.226	14.56	5.04
LS	1LS-1	5	60~77	90.09	−0.270	3.90	4.22
	1LS-2	9	59~63	89.50	−0.619	3.26	16.33
	1LS-3	9	74~75	103.81	−0.334	**2**.**66**	5.34
CLC	1CLC-1	3	7~9	5.57	0.473	3.79	10.49
	1CLC-2	3	70~90	66.42	0.622	8.31	20.14
	1CLC-3	5	15~17	16.06	−0.481	3.48	9.69
	1CLC-4	5	19~20	20.00	−0.433	**2**.**63**	7.29
	1CLC-5	5	25~29	26.31	−0.476	3.47	9.92
	1CLC-6	7	1~13	0.01	0.295	7.78	4.46
	1CLC-7	9	63~84	103.81	0.511	5.90	13.56
	1CLC-8	10	30~32	162.47	−1.304	**2**.**94**	16.29
LC	1LC-1	2	7~8	36.18	−0.423	**2**.**64**	7.78
	1LC-2	2	10	38.74	−0.436	3.04	9.27
	1LC-3	2	12~17	46.81	−0.473	**2**.**96**	10.84
	1LC-4	3	69~82	62.95	0.477	3.78	11.20
	1LC-5	7	4~28	12.20	0.570	8.97	15.31
	1LC-6	9	69~84	98.75	0.532	4.64	15.80
	1LC-7	9	87~90	118.66	0.482	3.68	12.55
LCL^*^	1LCL^*^-1	6	67~68	182.83	−1.160	3.12	8.85
	1LCL^*^-2	7	1~8	0.01	−1.298	4.16	11.28
	1LCL^*^-3	9	38~40	74.77	2.093	**2**.**67**	6.29
	1LCL^*^-4	9	77~87	111.39	−1.390	3.46	11.07
LCa^*^	1LCa^*^-1	10	32	212.47	21.375	21.38	80.38
LCb^*^	1LCb^*^-1	6	67~68	182.83	−1.906	4.53	17.01
	1LCb^*^-2	7	17~38	12.2	−1.266	4.03	7.12
	1LCb^*^-3	9	69~87	107.17	−2.477	6.47	22.56
CC	1CC-1	3	70~90	63.38	−0.336	4.55	13.50
	1CC-2	9	77~79	106.33	−0.313	3.35	11.45
	1CC-3	10	32	212.47	0.800	4.24	61.30
PC	1PC-1	1	1~11	5.1	−0.198	3.11	12.10
	1PC-2	7	1~10	1.74	−0.182	3.61	9.86
H	1H-1	3	57~61	48.24	−0.256	**2**.**87**	13.01
	1H-2	3	69~70	55.38	−0.274	**2**.**71**	13.51
	1H-3	8	23~25	72.64	−0.239	**2**.**71**	9.81
	1H-4	8	57~60	113.47	−0.222	3.52	9.96
BL	1BL-1	1	26~28	20.38	−4.709	**2**.**95**	9.95
	1BL-2	5	45~68	85.9	3.973	5.26	6.37
	1BL-3	6	36~51	107.95	−3.236	4.33	4.29

Note: PH, Plant Height; PD1, Plant Diameter 1; PD2, Plant Diameter 2; LL, Leaf Length; LWh, Leaf Width; PL, Petiole Length; PLL, Petiole With Leaf Length; LWg, Leaf Wing; LS, Leaf Shrink; CLC, Core Leaf Color; LC, Leaf Color; LCL*, Outer leaf color measured by CR-300 type colorimeter (from black to white); LCa*, Outer leaf color measured by CR-300 type colorimeter (from green to red); LCb*, Outer leaf color measured by CR-300 type colorimeter b (from blue to yellow); CC, inner and outer leaf Color Contrast; PC, Petiole Color; H, Heading; BL, Bolt Length. LG, linkage group; AE, Additive Effects; EV, Explained Variation. LOD values < 3 are marked in bold fonts.

**Table 4:** QTL identified in the F_2_ population of Chinese cabbage using QTLNetwork 2.1.

Traits	QTL name	LG	Bin interval	Position (cM)	AE	EV (%)
PD1	2PD1-1	10	27~28	103.2	2.9890	12.30
PL	2PL-1	6	48~49	107.9	−1.2022	14.28
PLL	2PLL-1	6	45~46	105.4	−2.0171	46.84
	2PLL-2	10	51~52	300.3	−2.5396	
LWg	2LWg-1	6	31~32	94.0	−0.3344	50.67
	2LWg-2	9	18~20	39.1	−0.1692	
	2LWg-3	10	50~51	299.9	−0.3702	
CLC	2CLC-1	3	81~82	66.4	0.5999	50.10
	2CLC-2	7	1~2	0.0	0.3747	
LC	2LC-1	9	90~91	118.6	0.3746	8.94
LCb^*^	2LCb^*^-1	7	29~30	22.6	−1.5485	27.08
	2LCb^*^-2	9	79~80	107.2	−1.6575	
CC	2CC-1	3	81~82	66.4	−0.3377	25.91
	2CC-2	9	78~79	106.3	−0.3051	
BL	2BL-1	5	59~60	86.3	5.5388	15.50

Note: PD1, Plant Diameter 1; PL, Petiole Length; PLL, Petiole With Leaf Length; LWg, Leaf Wing; CLC, Core Leaf Color; LC, Leaf Color; LCb*, Outer leaf color measured by CR-300 type colorimeter b (from blue to yellow); CC, inner and outer leaf Color Contrast; BL, Bolt Length. LG, linkage group; AE, Additive Effects; EV, Explained Variation.

**Table 5:** Epistasis effects for plant diameter and core leaf color in the F_2_ population of Chinese cabbage detected by QTLNetwork 2.1.

Traits	QTL_i	LG	Interval-i	QTL_j	LG	Interval-j
CLC	2CLC-3	5	38~39	2CLC-4	9	20~21
PD2	2PD2-1	3	16~17	2PD2-2	9	12~13

Note: CLC, Core Leaf Color; PD2, Plant Diameter 2. LG, linkage group.

**Table 6:** Common QTL detected by WinQTL cartographer 2.5 and QTLNetwork 2.1 in Chinese cabbage.

Traits	LG	WinQTL					QTLNetwork				
		QTL name	Bin interval	Position (cM)	EV (%)	AE	QTL name	Bin interval	Position (cM)	EV (%)	AE
PD1	10	1PD1−9	24~30	103.25	2.42	2.093	2PD1−1	27~28	103.2	12.3	2.989
PL	6	1PL-1	36~51	108.95	12.1	−1.126	2PL-1	48~49	107.9	14.28	−1.2022
PLL	10	1PLL-4	44~54	300.33	7.88	−1.603	2PLL-2	51~52	300.3	—	−2.5396
LWg	10	1LWg-5	42~54	299.65	5.04	−0.226	2LWg-3	50~51	299.9	—	−0.3702
CLC	3	1CLC-2	70~90	66.42	20.14	0.622	2CLC-1	81~82	66.4	50.1	0.5999
	7	1CLC-6	1~13	0.01	4.46	0.295	2CLC-2	1~2	0		0.3747
LC	9	1LC-7	87~90	118.66	8.85	−1.16	2LC-1	90~91	118.6	8.94	0.3746
LCb^*^	9	1LCb^*^-3	69~87	107.17	22.56	−2.477	2LCb^*^-2	79~80	107.2	—	−1.6575
CC	3	1CC-1	70~90	63.38	13.5	−0.336	2CC-1	81~82	66.4	25.91	−0.3377
	9	1CC-2	77~79	106.33	11.45	−0.313	2CC-2	78~79	106.3		−0.3051
BL	5	1BL-2	45~68	85.9	6.37	3.973	2BL-1	59~60	86.3	15.5	5.5388

Note: PD1, Plant Diameter 1; PL, Petiole Length; PLL, Petiole With Leaf Length; LWg, Leaf Wing; CLC, Core Leaf Color; LC, Leaf Color; LCb*, Outer leaf color measured by CR-300 type colorimeter b (from blue to yellow); CC, inner and outer leaf Color Contrast; BL, Bolt Length. LG, linkage group; AE, Additive Effects; EV, Explained Variation.
