# Supplementary material for: A genome-wide SNP-based genetic map and QTL mapping for agronomic traits in Chinese cabbage
Source: Sci Rep. 2017 Apr 18;7:46305. doi: 10.1038/srep46305 (PMC5394690; doi:10.1038/srep46305)
Supplement: Supplementary Dataset 1 [file srep46305-s1.doc]

**A** **genome-wide SNP-based genetic map and QTL mapping for agronomic traits in Chinese cabbage**

Li Huang, Yafei Yang, Fang Zhang, Jiashu Cao

*1 Laboratory of Cell & Molecular Biology,* *Institute of Vegetable Science, Zhejiang University, Hangzhou 310058, China*

*2 Key Laboratory of Horticultural Plant Growth, Development and Quality Improvement, Ministry of Agriculture, Hangzhou 310058, China*

*3 Zhejiang Provincial Key Laboratory of Horticultural Plant Integrative Biology Hangzhou 310058, China*

**Supplementary Figures**

(B)


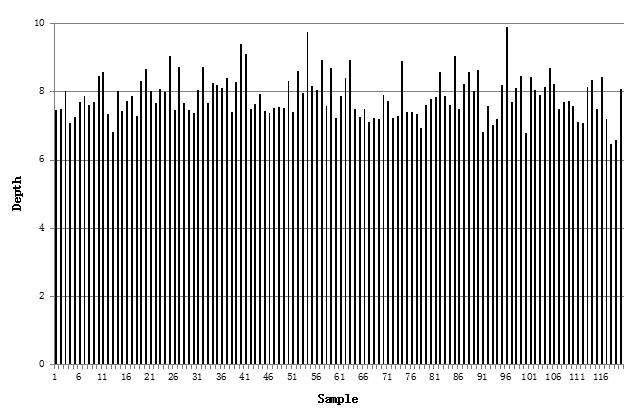


(A)

Sample


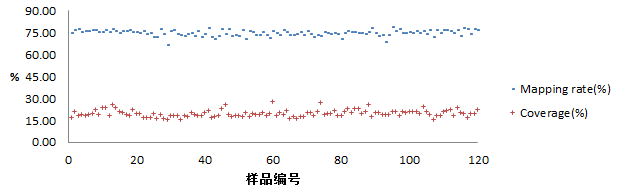


mapping rate

coverage

（%）


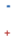


Sample

Depth

Fig. S1 The alignment of clean reads from the F_2_ individuals of Chinese cabbage with the reference genome and the average sequencing depth at the SNP positions in the F_2_ individuals. (A) The mapping rate of clean reads from 120 F_2_ individuals with the reference genome and the percentage of the genome covered by reads. (B) The average sequencing depth at the SNP positions in 120 F_2_ individuals.


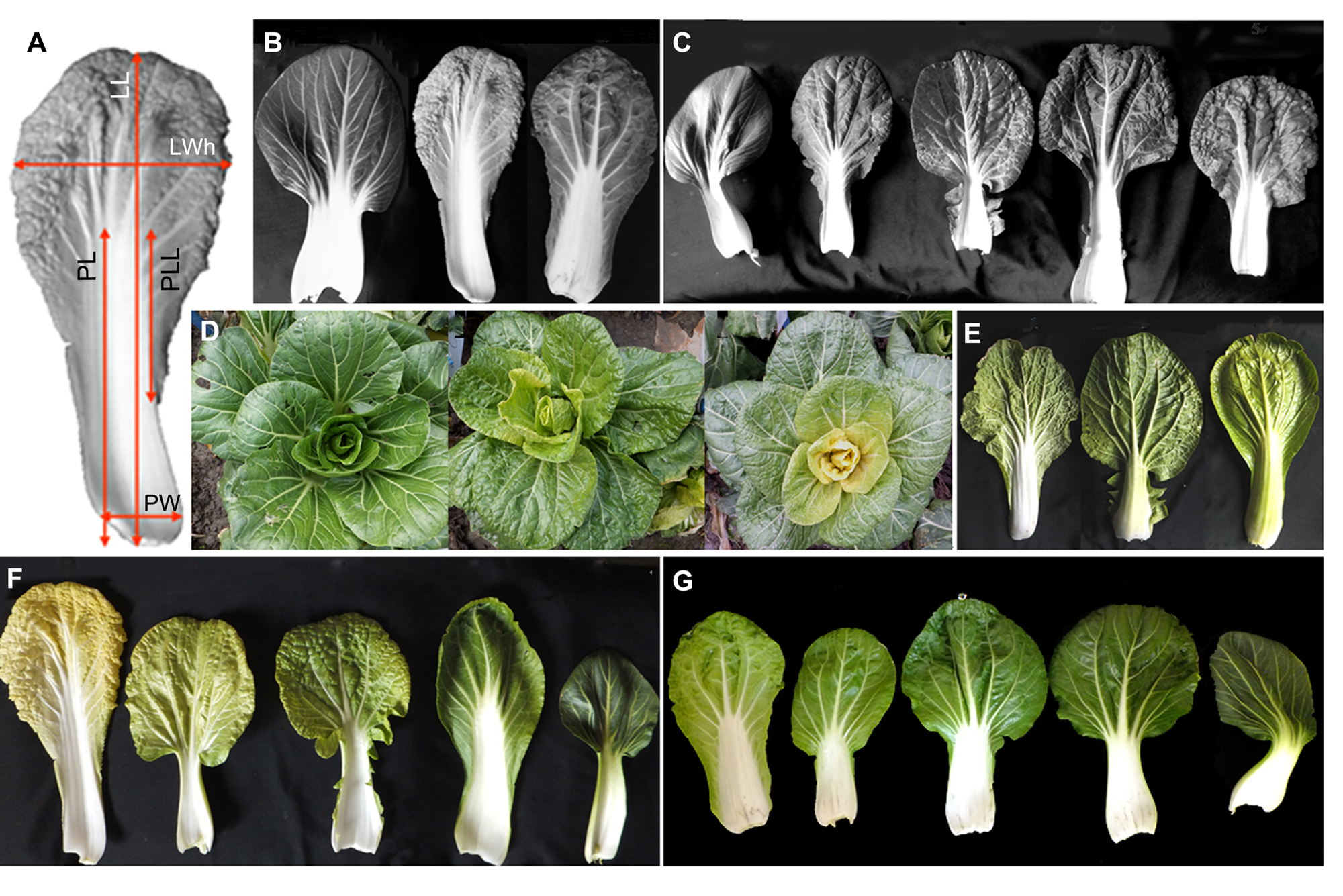
**Fig. S2 Measurement or classification criteria of some traits** in **Chinese cabbage**. (A) The measurement criterion of leaf length and width; LL, Leaf Length; LW, leaf Width; PL, Petiole Length; PLL, Petiole with Leaf Length; PW, Petiole Width; (B) The classification criterion of leaf wing, recording as 1-3 from left to right respectively; (C) The classification criterion of leaf shrink, recording as 1-5 from left to right respectively; (D) The classification criterion of inner and outer leaf color contrast, recording as 1-3 from left to right respectively; (E) The classification criterion of petiole color, recording as 1-3 from left to right respectively; (F) The classification criterion of core leaf color, recording as 1-5 from left to right respectively; (G) The classification criterion of leaf color, recording as 1-5 from left to right respectively.
